# The effects of pre versus post workout supplementation of creatine monohydrate on body composition and strength

**DOI:** 10.1186/1550-2783-10-S1-P1

**Published:** 2013-12-06

**Authors:** Victoria Ciccone, Kristina Cabrera, Jose Antonio

**Affiliations:** 1 Exercise and Sports Sciences, Nova Southeastern University, 3532 S. University Drive, University Park Plaza Suite 3532, Davie FL 33314, USA

## Background

Chronic supplementation with creatine monohydrate has been shown to promote increases in intramuscular total creatine, phosphocreatine, skeletal muscle mass, lean body mass and muscle fiber size. Furthermore, there is robust evidence that muscular strength and power will also increase after supplementing with creatine. However, it is not known if the timing of creatine supplementation will affect the adaptive response to exercise. Thus, the purpose of this investigation was to determine the difference between pre versus post exercise supplementation of creatine on measures of body composition and strength.

## Methods

Nineteen healthy recreational male bodybuilders (age: 22.87+2.90; height: 172.69+13.39cm; weight: 80.18+10.43kg) participated in this study. Subjects were randomly assigned to one of the following groups: PRE-SUPP or POST-SUPP workout supplementation of creatine (5 grams). The PRE-SUPP group consumed 5 grams of creatine immediately before exercise. On the other hand, the POST-SUPP group consumed 5 grams immediately after exercise. Subjects trained on average five days per week for four weeks. Subjects consumed the supplement on the two non-training days at their convenience. Subjects performed a periodized, split-routine, bodybuilding workout five days per week (Chest-shoulders-triceps; Back-biceps, Legs, etc). Body composition (Bod Pod®) and 1-RM bench press were determined. Diet logs were collected and analyzed (one random day per week; four total days analyzed).

## Results

2x2 ANOVA results - There was a significant time effect for FFW (F=19.9; p=0.001) and BP (F=18.9; p<0.001), however FM and BW did not reach significance. While there were trends, no significant interactions were found.

**Table 1:** Body composition and strength

		Baseline	Post-Test	Change
**POST-SUPP**	BW (kg)	78.05+10.41	78.85+9.97	0.80+0.85

**PRE-SUPP**		82.87+9.99	82.87+10.62	0.33+2.17

**POST-SUPP**	FFM (kg)	65.89+8.02	67.91+8.56	2.02+1.17

**PRE-SUPP**		66.38+6.54	67.57+7.62	0.88+1.84

**POST-SUPP**	Fat Mass (kg)	12.98+4.00	11.75+3.58	-1.23+1.60

**PRE-SUPP**		16.08+5.06	15.30+5.53	-0.11+2.00

**POST-SUPP**	% Body Fat	16.89+4.79	14.97+4.65	-1.92+2.25

**PRE-SUPP**		19.09+4.54	18.17+5.13	-0.17+2.2

**POST-SUPP**	1-RM BP	103.16+23.99	110.91+25.35	7.75+6.16

**PRE-SUPP**		95.45+21.02	103.28+19.49	6.57+8.15

Values are mean+SD. 1-RM – one repetition maximum; BP – Bench Press BW – body weight; FFM – fat-free mass

**Table 2:** Magnitude-based inference results

	POST-SUPP	PRE-SUPP		
**Measures**	Mean+SD	Mean+SD	Difference+90CI^a^	Qualitative Inference

**BW (kg)**	0.8+0.9	0.3+2.2	0.5+1.3	Trivial

**FFM (kg)**	2.0+1.2	0.9+1.8	1.1+1.2	Possibly beneficial

**FM (kg)**	-1.2+1.6	-0.1+2.0	1.1+1.5	Possibly beneficial

**Bench Press 1-RM (kg)**	7.6+6.1	6.6+8.2	1.2+1.7	Likely beneficial

Changes in body composition and performance in PRE-SUPP vs. POST-SUPP groups, and qualitative inferences about the effects on body composition and bench press strength

Values reported as mean + standard deviation (SD); BW – body weight; FFM – fat-free mass; FM – fat mass. ^a^+90%CI: add and subtract this number to the mean difference to obtain the 90% confidence intervals for the true difference. Qualitative inference represents the likelihood that the true value will have the observed magnitude. Furthermore, there were no differences in caloric or macronutrient intake between the groups.

## Conclusion

Creatine supplementation plus resistance exercise increases fat-free mass and strength. Based on the magnitude inferences it appears that consuming creatine immediately post-workout is superior to pre-workout vis a vis body composition and strength.

